# Supplementary material for: A carbon-nitrogen negative feedback loop underlies the repeated evolution of cnidarian–Symbiodiniaceae symbioses
Source: Nat Commun. 2023 Nov 1;14:6949. doi: 10.1038/s41467-023-42582-y (PMC10620218; doi:10.1038/s41467-023-42582-y)
Supplement: Supplementary file 1 — Supplementary Information [file 41467_2023_42582_MOESM1_ESM.pdf]

Supplementary Information for

A carbon-nitrogen negative feedback loop underlies the repeated evolution of cnidarian-Symbiodiniaceae symbioses

Guoxin Cui[1]†*, Jianing Mi[2,3]†, Alessandro Moret[1]†, Jessica Menzies[1], Huawen Zhong[1], Angus Li[1], Shiou-Han Hung[1], Salim Al-Babili[2,4], Manuel Aranda[1]*

*Corresponding author. Guoxin Cui (guoxin.cui@kaust.edu.sa) and Manuel Aranda (manuel.aranda@kaust.edu.sa)

This PDF file includes:

Supplementary Figures 1 to 27

Supplementary Table 1

Other Supplementary Materials for this manuscript includes:

Supplementary Data 1 to 5

Supplementary Figures

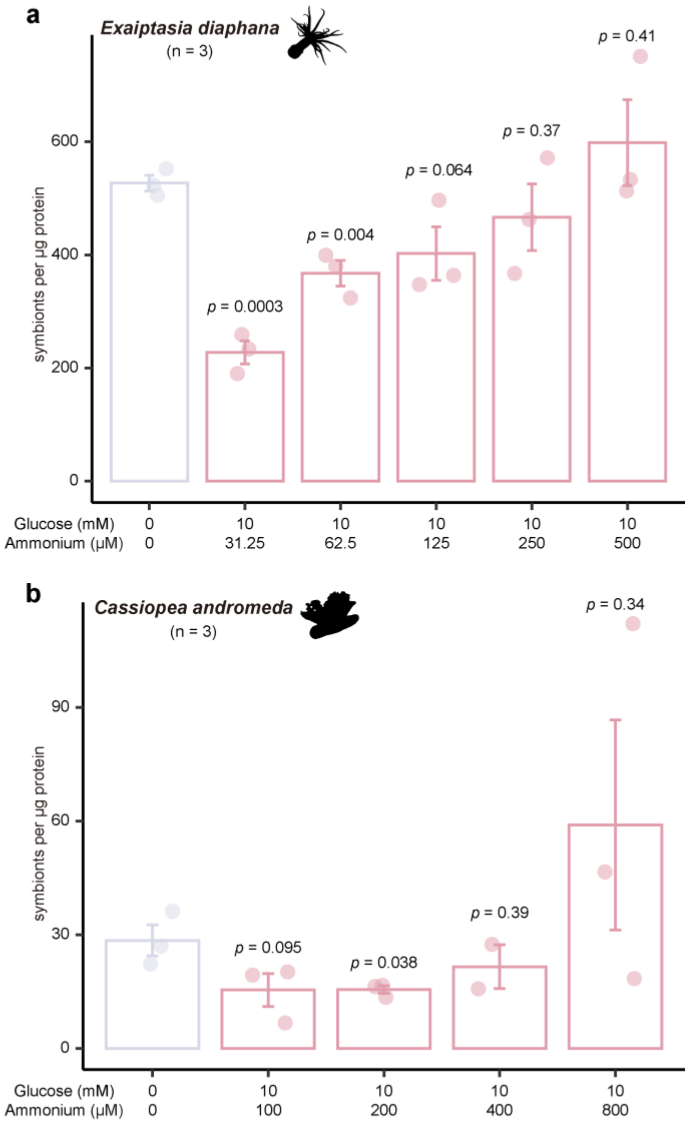

Supplementary Figure 1 Restoring glucose-induced symbiont loss with increased ammonium concentrations in E. diaphana (a) and C. andromeda (b), respectively.

p values were calculated using two-sided Welch's *t* tests between the appropriate condition and the control in each experiment. n indicates the number of biologically independent animals used in each experiment.

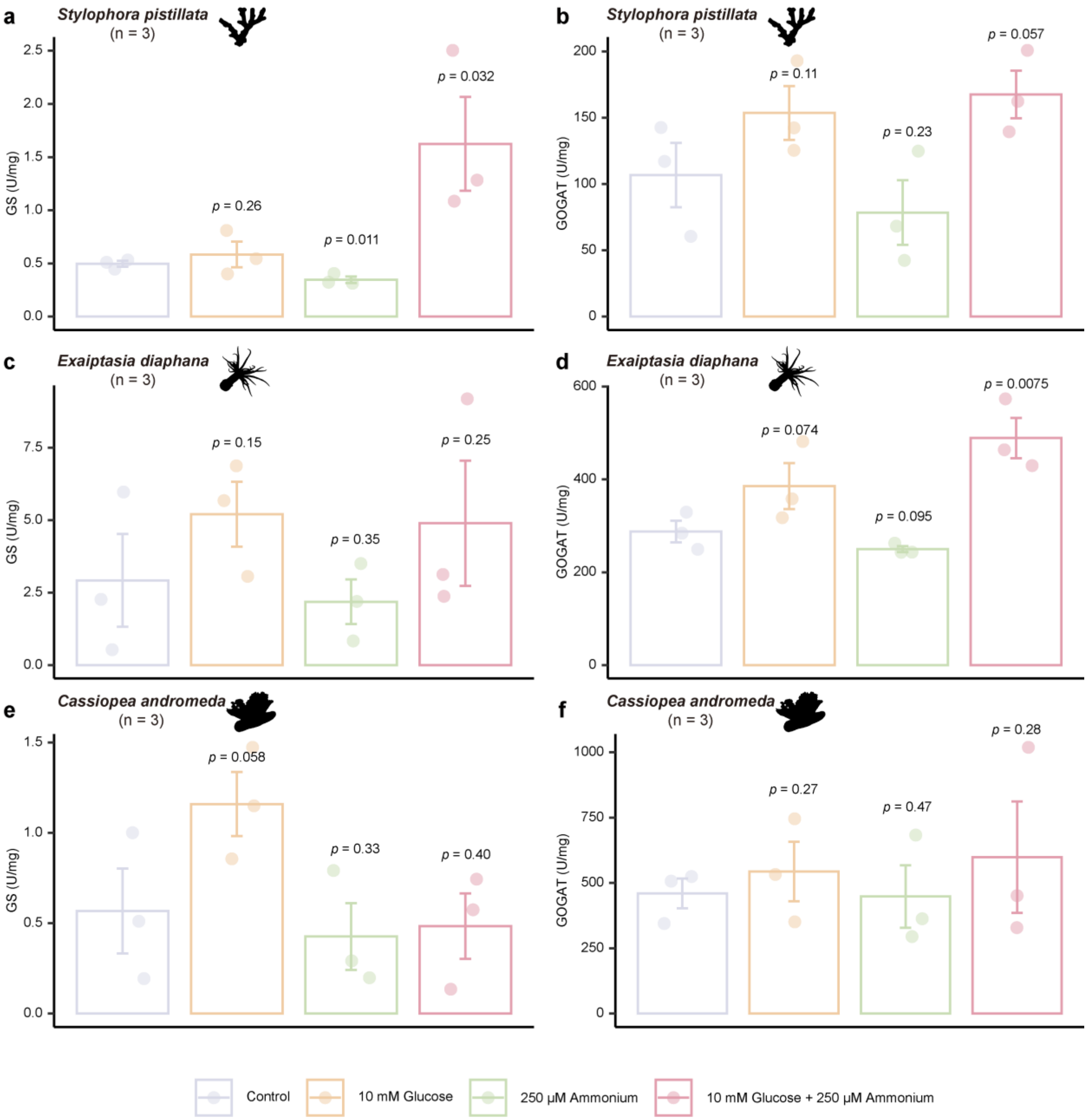

Supplementary Figure 2 Changes of GS (a, c, e) and GOGAT (b, d, f) enzyme activities in response to nutrient supplementations in *S. pistillata* (a, b), *E. diaphana* (c, d), and *C. andromeda* (e, f).

One unit of enzyme activity was defined as the amount of enzyme producing 1 nmol of ADP (for GS) or decomposing 1 nmol of NADH (for GOGAT) per minute at pH 7.5 at 26 °C. *p* values were calculated using two-sided Welch's *t* tests between the appropriate condition and the control in each experiment. n indicates the number of biologically independent animals used in each experiment.

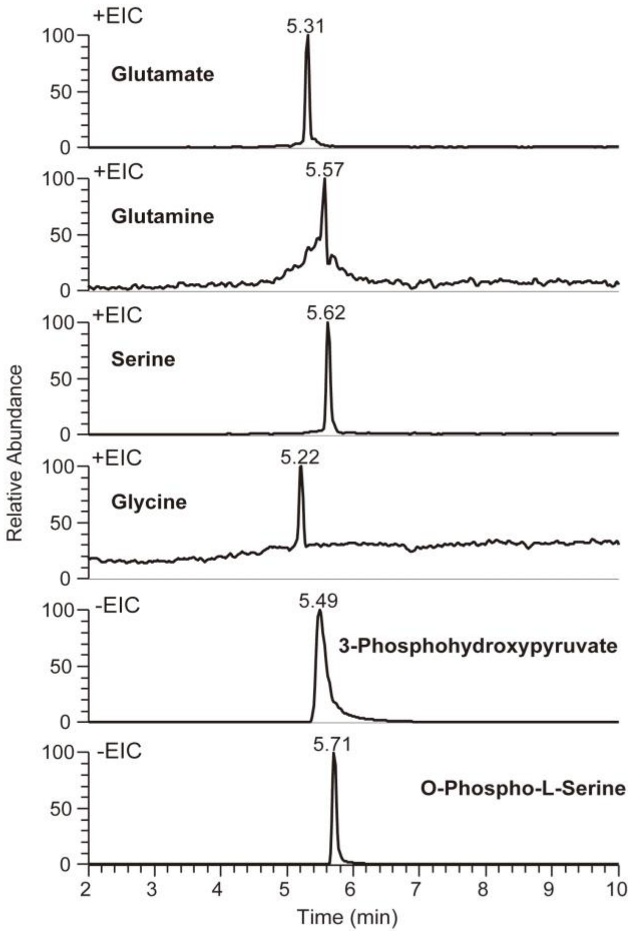

Supplementary Figure 3. Extracted ion chromatograms (EIC) of standards.

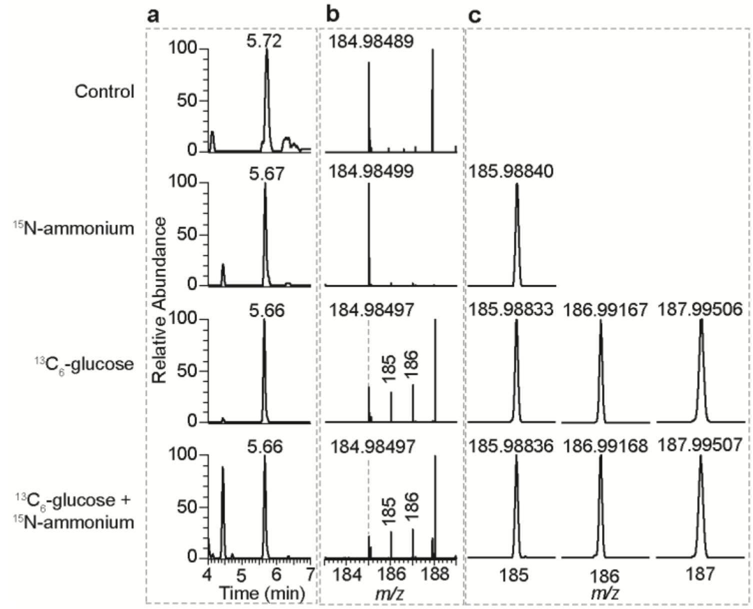

Supplementary Figure 4. Identification of 3-phosphohydroxypyruvate isolated from symbiotic *S. pistillata*.

(**a, b**) Extracted ion chromatograms (EIC) (**a**) and the isotopic distributions (**b**) of 3-phosphohydroxypyruvate isolated from symbiotic *S. pistillata* at different conditions. (**c**) The zoom of the area in (b) showing that different isotopologue compositions of 3-phosphohydroxypyruvate are distinguished by HR-MS.

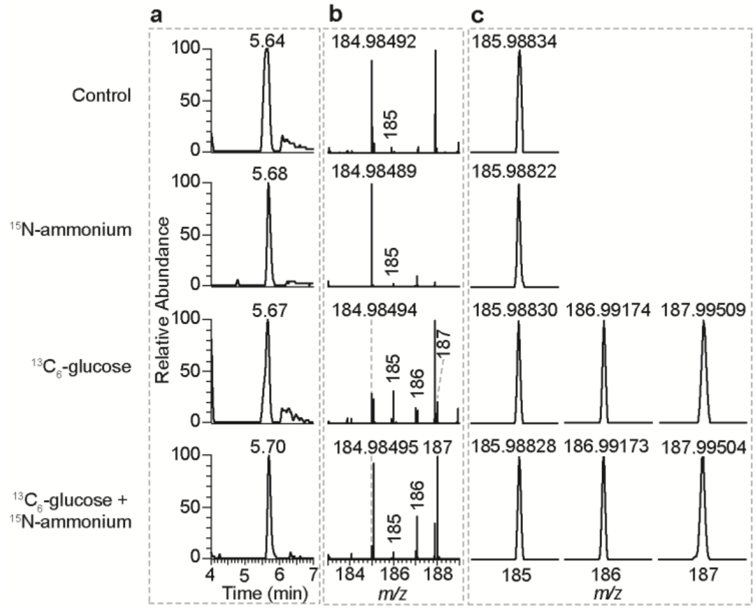

Supplementary Figure 5. Identification of 3-phosphohydroxypyruvate isolated from symbiotic *C. andromeda*.

(**a, b**) Extracted ion chromatograms (EIC) (**a**) and the isotopic distributions (**b**) of 3-phosphohydroxypyruvate isolated from symbiotic *C. andromeda* at different conditions. (**c**) The zoom of the area in (b) showing that different isotopologue compositions of 3-phosphohydroxypyruvate are distinguished by HR-MS.

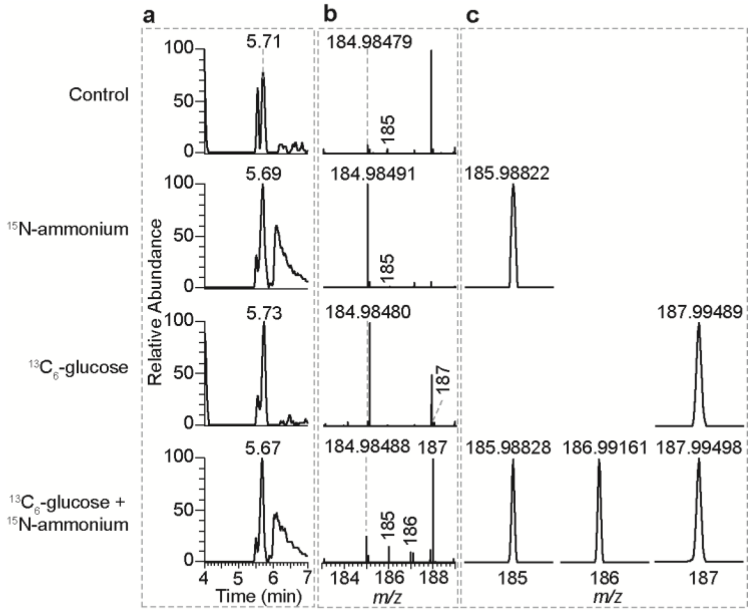

Supplementary Figure 6. Identification of 3-phosphohydroxypyruvate isolated from symbiotic *E. diaphana*.

(**a, b**) Extracted ion chromatograms (EIC) (**a**) and the isotopic distributions (**b**) of 3-phosphohydroxypyruvate isolated from symbiotic *E. diaphana* at different conditions. (**c**) The zoom of the area in (b) showing that different isotopologue compositions of 3-phosphohydroxypyruvate are distinguished by HR-MS.

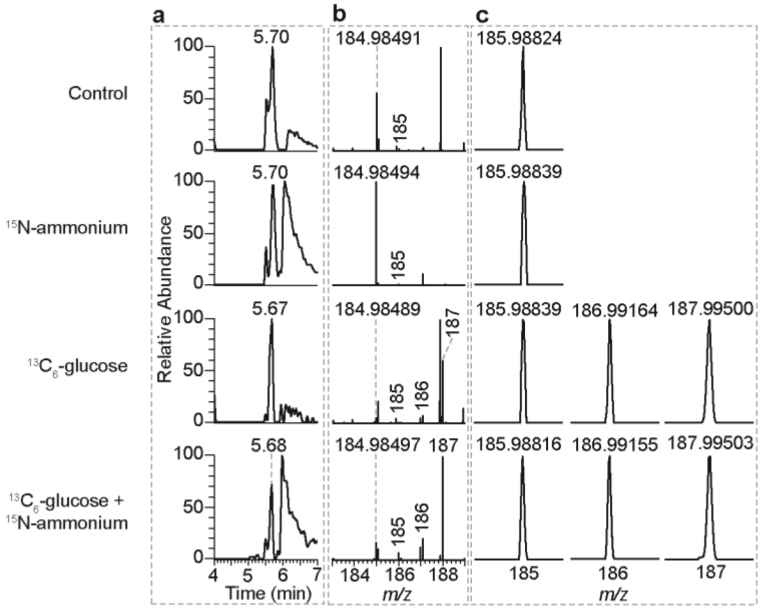

Supplementary Figure 7. Identification of 3-phosphohydroxypyruvate isolated from aposymbiotic *E. diaphana*.

(**a, b**) Extracted ion chromatograms (EIC) (**a**) and the isotopic distributions (**b**) of 3-phosphohydroxypyruvate isolated from aposymbiotic *E. diaphana* at different conditions. (**c**) The zoom of the area in (b) showing that different isotopologue compositions of 3-phosphohydroxypyruvate are distinguished by HR-MS.

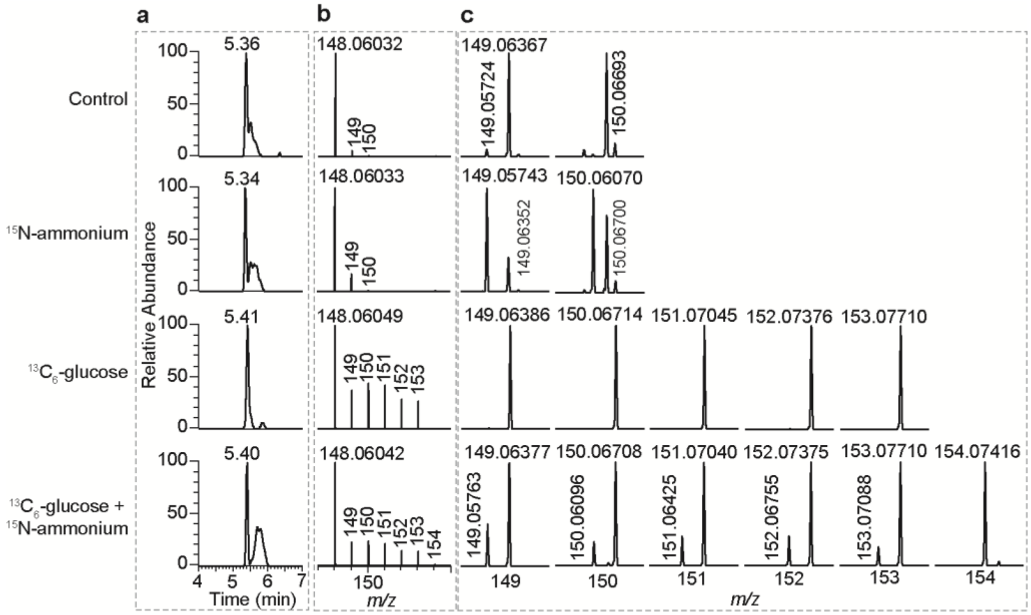

Supplementary Figure 8. Identification of glutamate isolated from symbiotic *S. pistillata*.

(**a, b**) Extracted ion chromatograms (EIC) (**a**) and the isotopic distributions (**b**) of glutamate isolated from symbiotic *S. pistillata* at different conditions. (**c**) The zoom of the area in (b) showing that different isotopologue compositions of glutamate are distinguished by HR-MS.

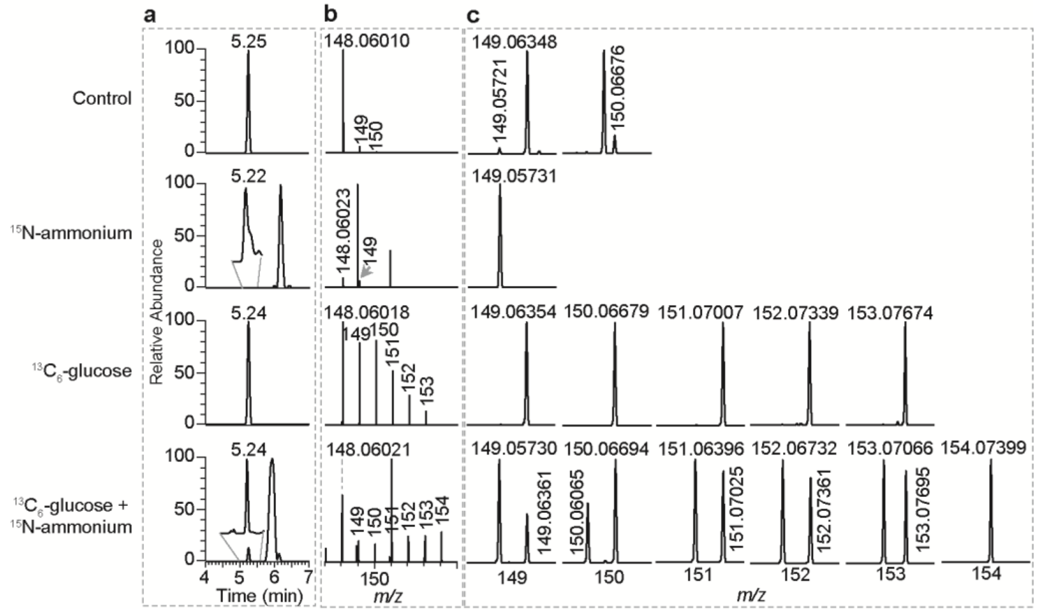

Supplementary Figure 9. Identification of glutamate isolated from symbiotic *C. andromeda*.

(**a, b**) Extracted ion chromatograms (EIC) (**a**) and the isotopic distributions (**b**) of glutamate isolated from symbiotic *C. andromeda* at different conditions. (**c**) The zoom of the area in (b) showing that different isotopologue compositions of glutamate are distinguished by HR-MS.

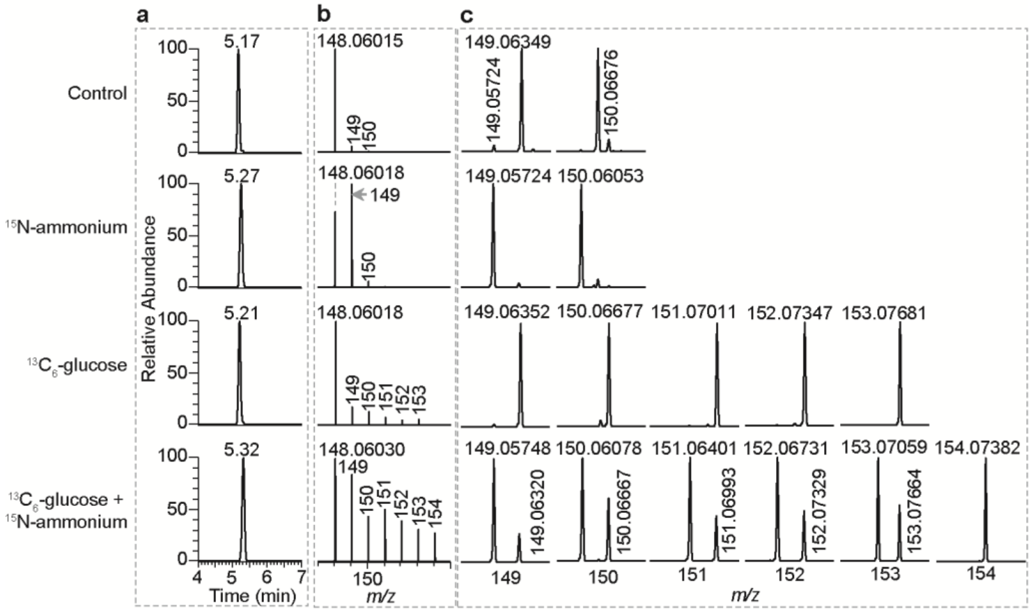

Supplementary Figure 10. Identification of glutamate isolated from symbiotic *E. diaphana*.

(**a, b**) Extracted ion chromatograms (EIC) (**a**) and the isotopic distributions (**b**) of glutamate isolated from symbiotic *E. diaphana* at different conditions. (**c**) The zoom of the area in (b) showing that different isotopologue compositions of glutamate are distinguished by HR-MS.

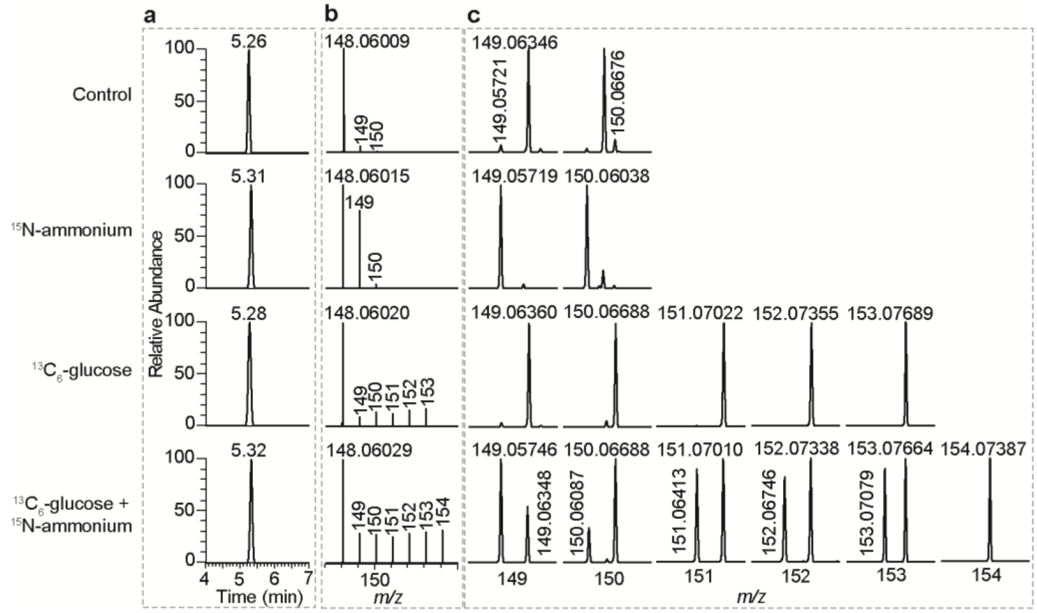

Supplementary Figure 11. Identification of glutamate isolated from aposymbiotic *E. diaphana*.

(**a, b**) Extracted ion chromatograms (EIC) (**a**) and the isotopic distributions (**b**) of glutamate isolated from aposymbiotic *E. diaphana* at different conditions. (**c**) The zoom of the area in (b) showing that different isotopologue compositions of glutamate are distinguished by HR-MS.

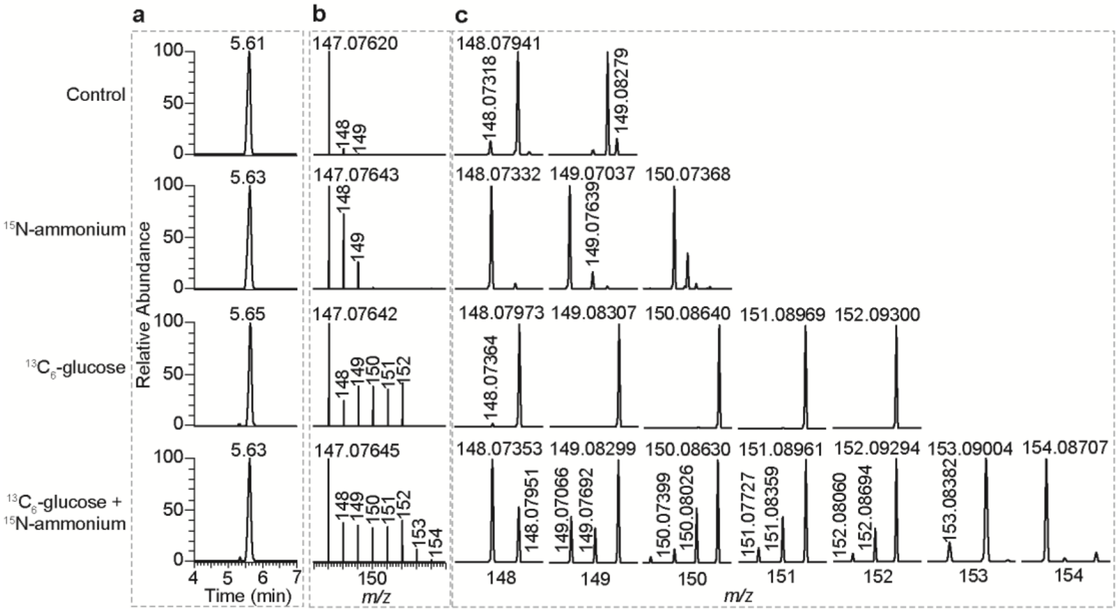

Supplementary Figure 12. Identification of glutamine isolated from symbiotic *S. pistillata*.

(**a, b**) Extracted ion chromatograms (EIC) (**a**) and the isotopic distributions (**b**) of glutamine isolated from symbiotic *S. pistillata* at different conditions. (**c**) The zoom of the area in (b) showing that different isotopologue compositions of glutamine are distinguished by HR-MS.

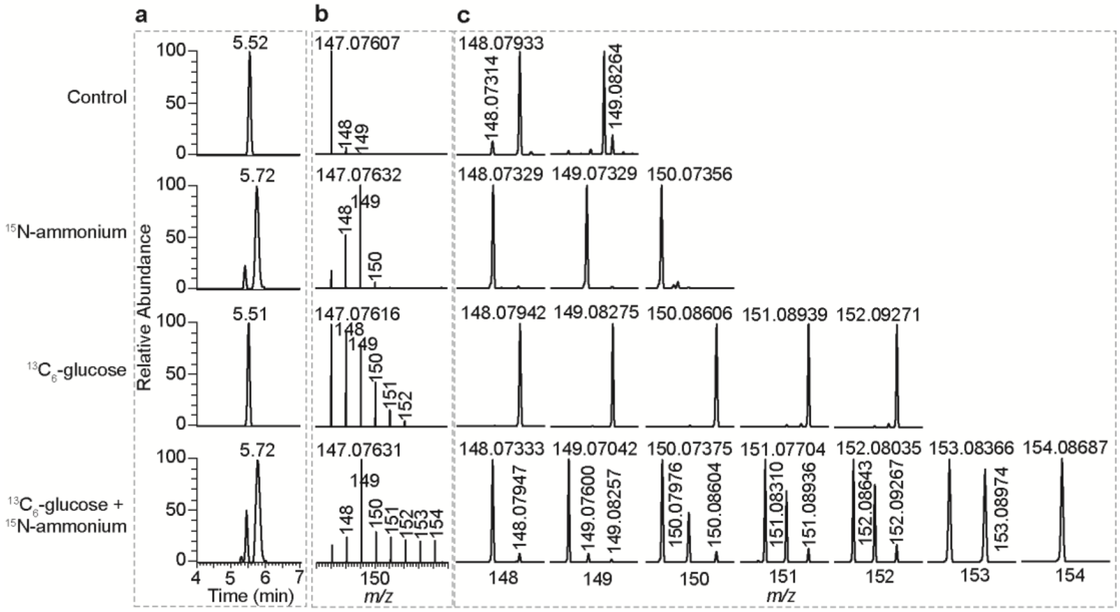

Supplementary Figure 13. Identification of glutamine isolated from symbiotic *C. andromeda*.

(**a, b**) Extracted ion chromatograms (EIC) (**a**) and the isotopic distributions (**b**) of glutamine isolated from symbiotic *C. andromeda* at different conditions. (**c**) The zoom of the area in (b) showing that different isotopologue compositions of glutamine are distinguished by HR-MS.

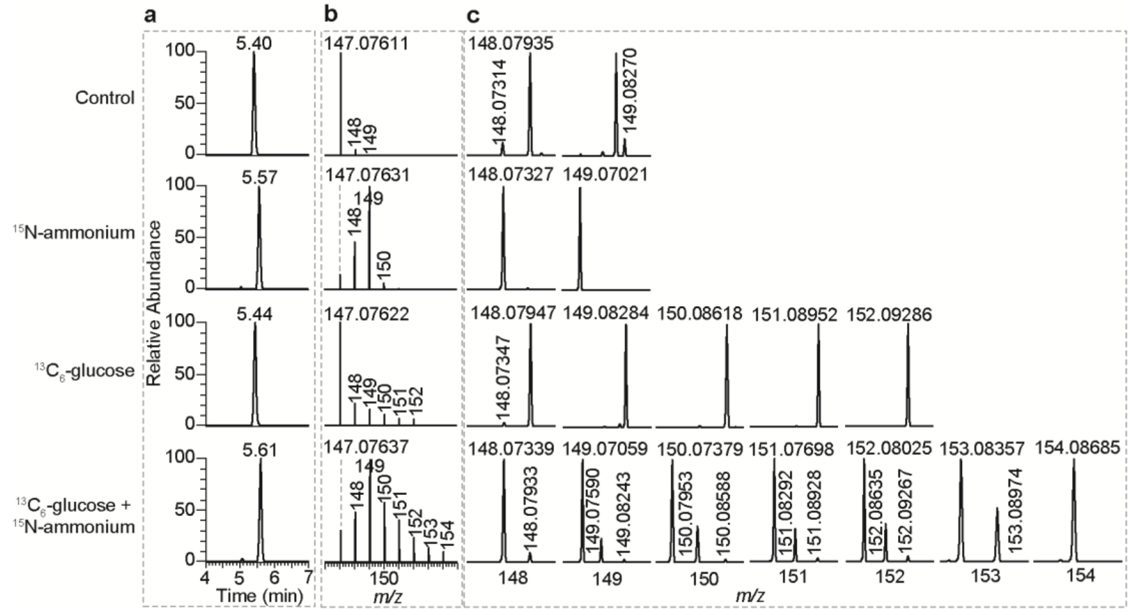

Supplementary Figure 14. Identification of glutamine isolated from symbiotic *E. diaphana*.

(**a, b**) Extracted ion chromatograms (EIC) (**a**) and the isotopic distributions (**b**) of glutamine isolated from symbiotic *E. diaphana* at different conditions. (**c**) The zoom of the area in (b) showing that different isotopologue compositions of glutamine are distinguished by HR-MS.

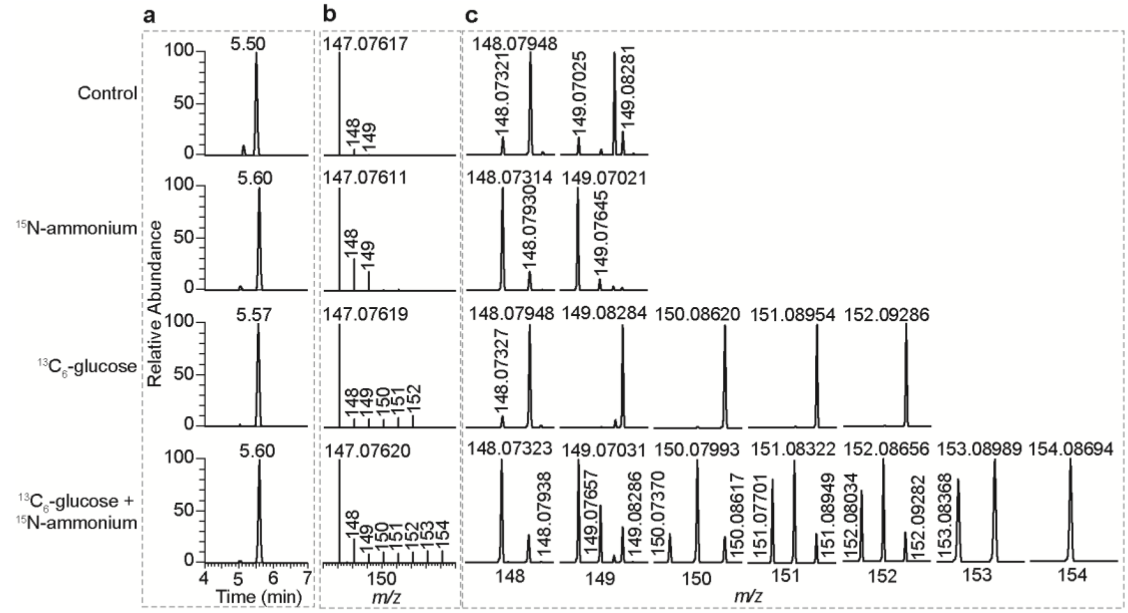

Supplementary Figure 15. Identification of glutamine isolated from aposymbiotic *E. diaphana*.

(**a, b**) Extracted ion chromatograms (EIC) (**a**) and the isotopic distributions (**b**) of glutamine isolated from aposymbiotic *E. diaphana* at different conditions. (**c**) The zoom of the area in (b) showing that different isotopologue compositions of glutamine are distinguished by HR-MS.

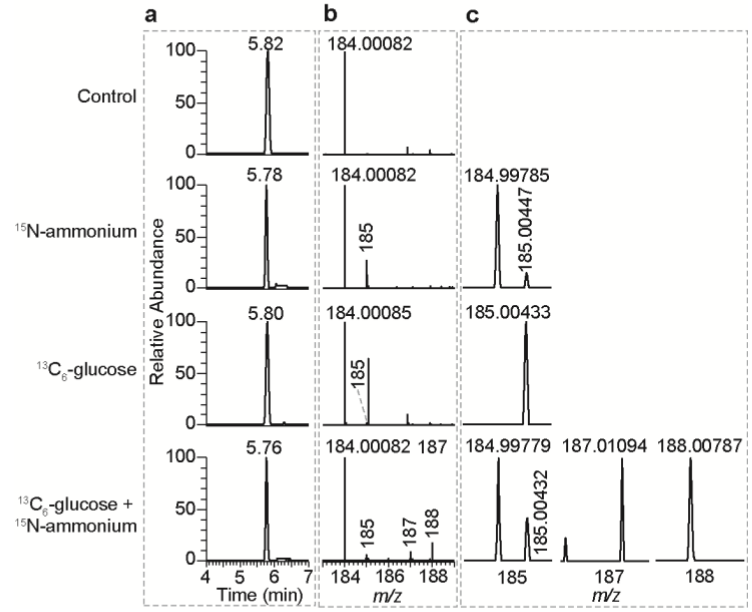

Supplementary Figure 16. Identification of O-phospho-*L*-serine isolated from symbiotic *E. diaphana*. (**a, b**) Extracted ion chromatograms (EIC) (**a**) and the isotopic distributions (**b**) of O-phospho-*L*-serine isolated from symbiotic *E. diaphana* at different conditions. (**c**) The zoom of the area in (b) showing that different isotopologue compositions of O-phospho-*L*-serine are distinguished by HR-MS.

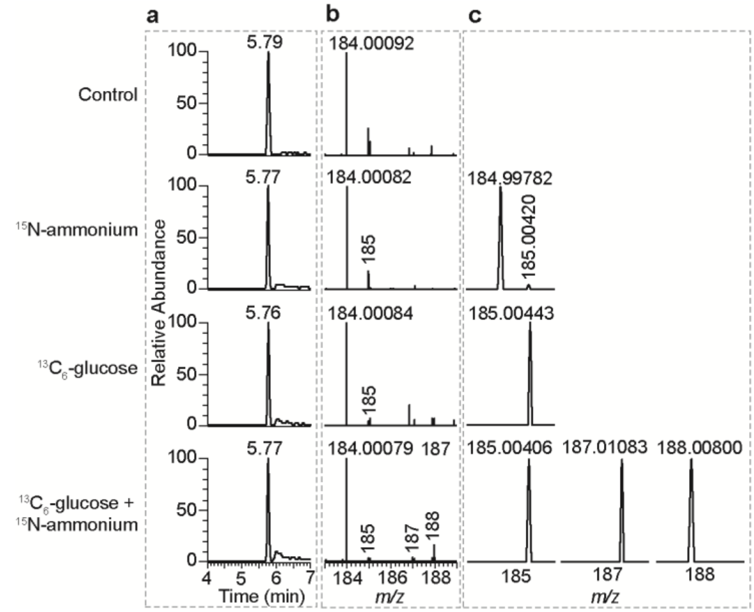

Supplementary Figure 17. Identification of O-phospho-*L*-serine isolated from aposymbiotic *E. diaphana*.

(**a, b**) Extracted ion chromatograms (EIC) (**a**) and the isotopic distributions (**b**) of O-phospho-*L*-serine isolated from aposymbiotic *E. diaphana* at different conditions. (**c**) The zoom of the area in (b) showing that different isotopologue compositions of O-phospho-*L*-serine are distinguished by HR-MS.

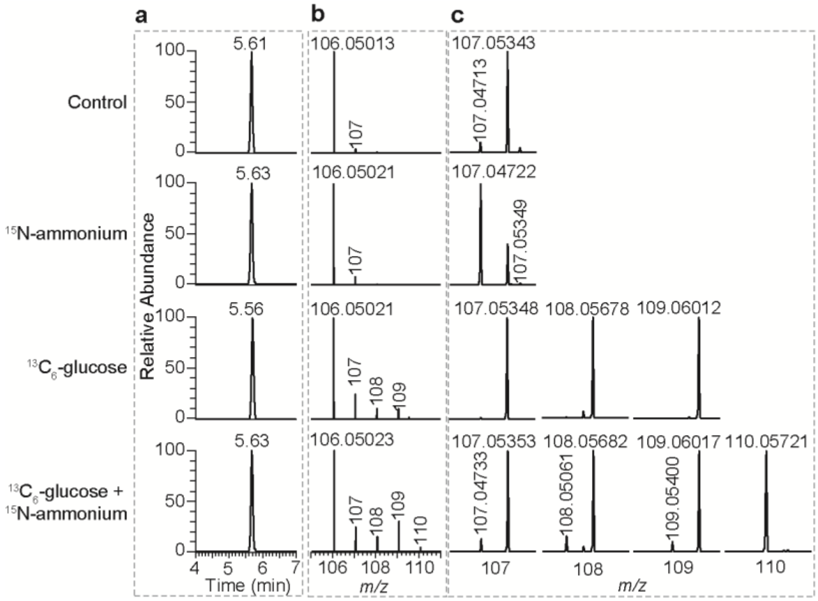

Supplementary Figure 18. Identification of serine isolated from symbiotic *S. pistillata*.

(**a, b**) Extracted ion chromatograms (EIC) (**a**) and the isotopic distributions (**b**) of serine isolated from symbiotic *S. pistillata* at different conditions. (**c**) The zoom of the area in (b) showing that different isotopologue compositions of serine are distinguished by HR-MS.

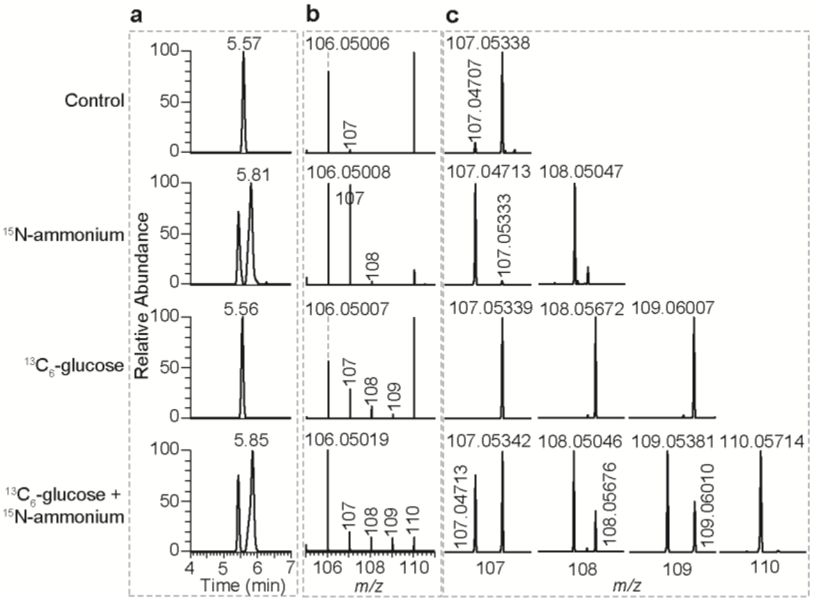

Supplementary Figure 19. Identification of serine isolated from symbiotic *C. andromeda*.

(**a, b**) Extracted ion chromatograms (EIC) (**a**) and the isotopic distributions (**b**) of serine isolated from symbiotic *C. andromeda* at different conditions.

(**c**) The zoom of the area in (b) showing that different isotopologue compositions of serine are distinguished by HR-MS.

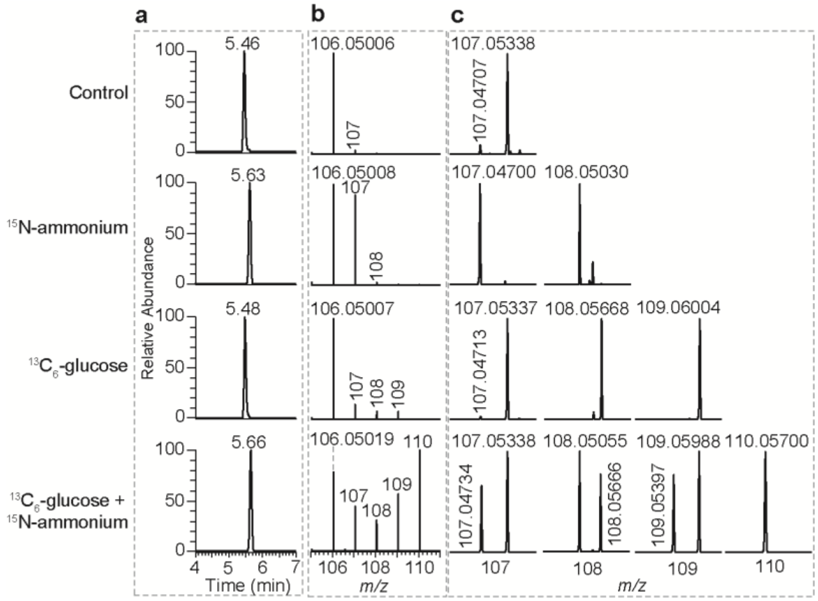

Supplementary Figure 20. Identification of serine isolated from symbiotic *E. diaphana*.

(**a, b**) Extracted ion chromatograms (EIC) (**a**) and the isotopic distributions (**b**) of serine isolated from symbiotic *E. diaphana* at different conditions. (**c**) The zoom of the area in (b) showing that different isotopologue compositions of serine are distinguished by HR-MS.

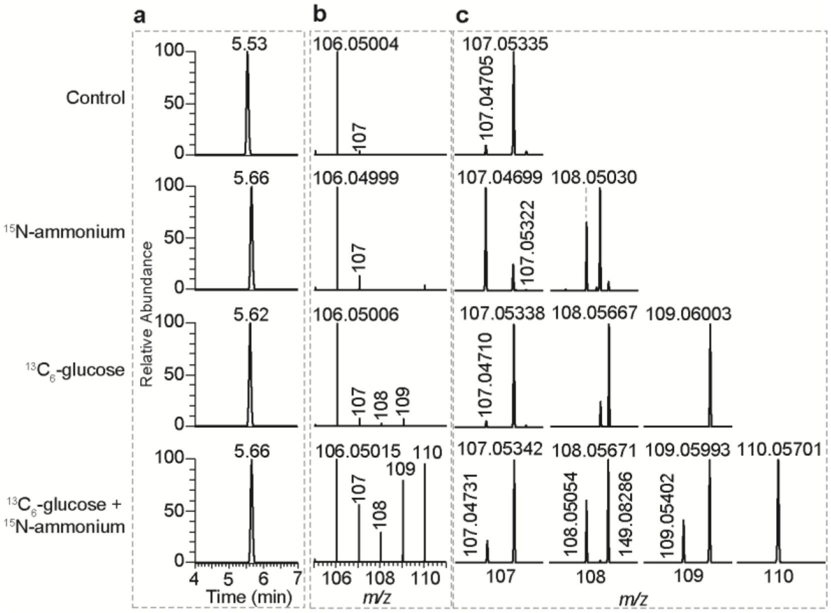

Supplementary Figure 21. Identification of serine isolated from aposymbiotic *E. diaphana*.

(**a, b**) Extracted ion chromatograms (EIC) (**a**) and the isotopic distributions (**b**) of serine isolated from aposymbiotic *E. diaphana* at different conditions. (**c**) The zoom of the area in (b) showing that different isotopologue compositions of serine are distinguished by HR-MS.

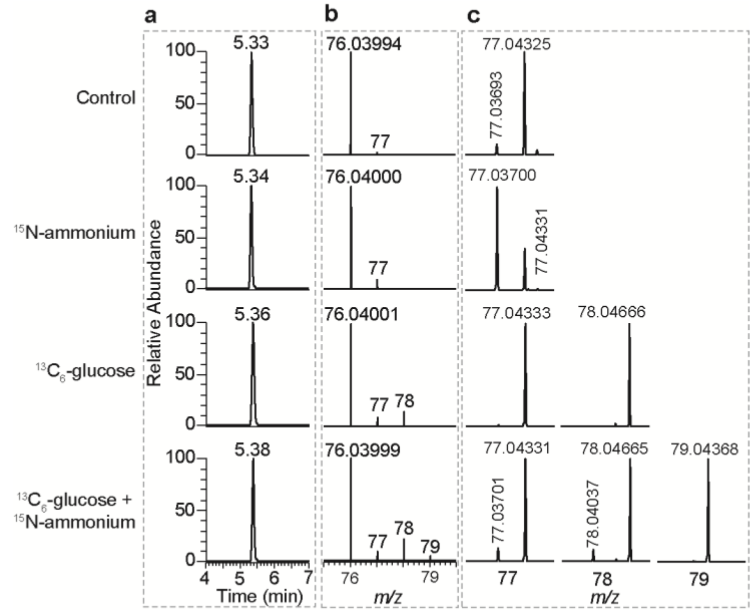

Supplementary Figure 22. Identification of glycine isolated from symbiotic *S. pistillata*.

(**a, b**) Extracted ion chromatograms (EIC) (**a**) and the isotopic distributions (**b**) of glycine isolated from symbiotic *S. pistillata* at different conditions. (**c**) The zoom of the area in (b) showing that different isotopologue compositions of glycine are distinguished by HR-MS.

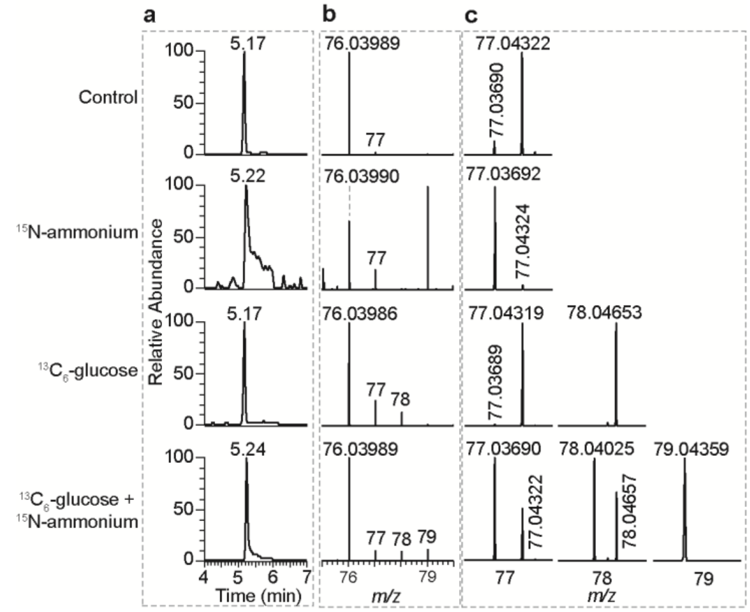

Supplementary Figure 23. Identification of glycine isolated from symbiotic *C. andromeda*.

(**a, b**) Extracted ion chromatograms (EIC) (**a**) and the isotopic distributions (**b**) of glycine isolated from symbiotic *C. andromeda* at different conditions. (**c**) The zoom of the area in (b) showing that different isotopologue compositions of glycine are distinguished by HR-MS.

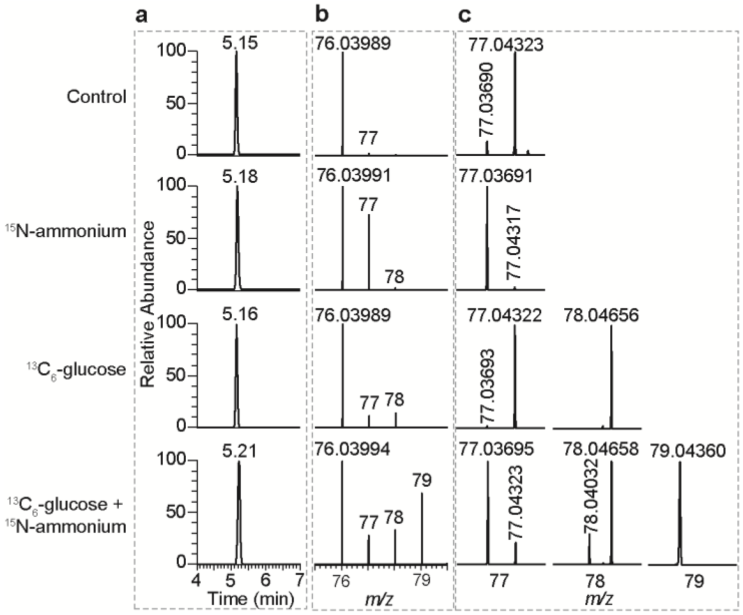

Supplementary Figure 24. Identification of glycine isolated from symbiotic *E. diaphana*.

(**a, b**) Extracted ion chromatograms (EIC) (**a**) and the isotopic distributions (**b**) of glycine isolated from symbiotic *E. diaphana* at different conditions. (**c**) The zoom of the area in (b) showing that different isotopologue compositions of glycine are distinguished by HR-MS.

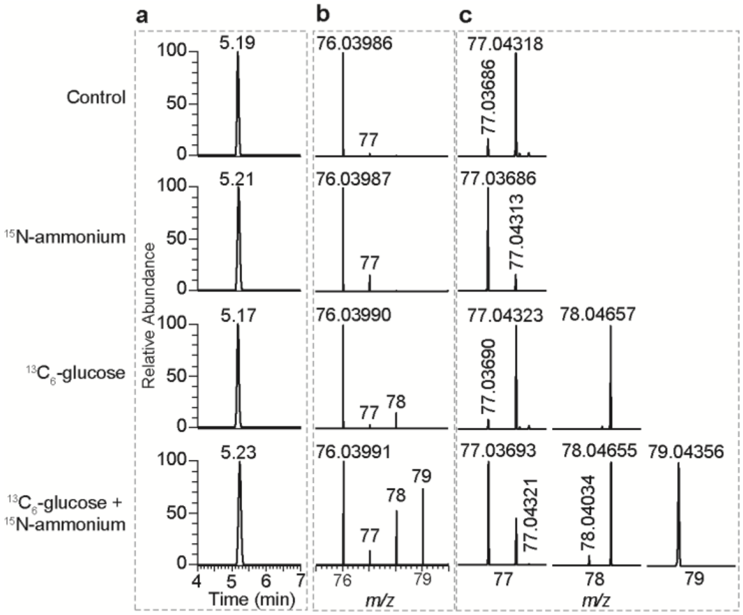

Supplementary Figure 25. Identification of glycine isolated from aposymbiotic *E. diaphana*.

(**a, b**) Extracted ion chromatograms (EIC) (**a**) and the isotopic distributions (**b**) of glycine isolated from aposymbiotic *E. diaphana* at different conditions. (**c**) The zoom of the area in (b) showing that different isotopologue compositions of glycine are distinguished by HR-MS.

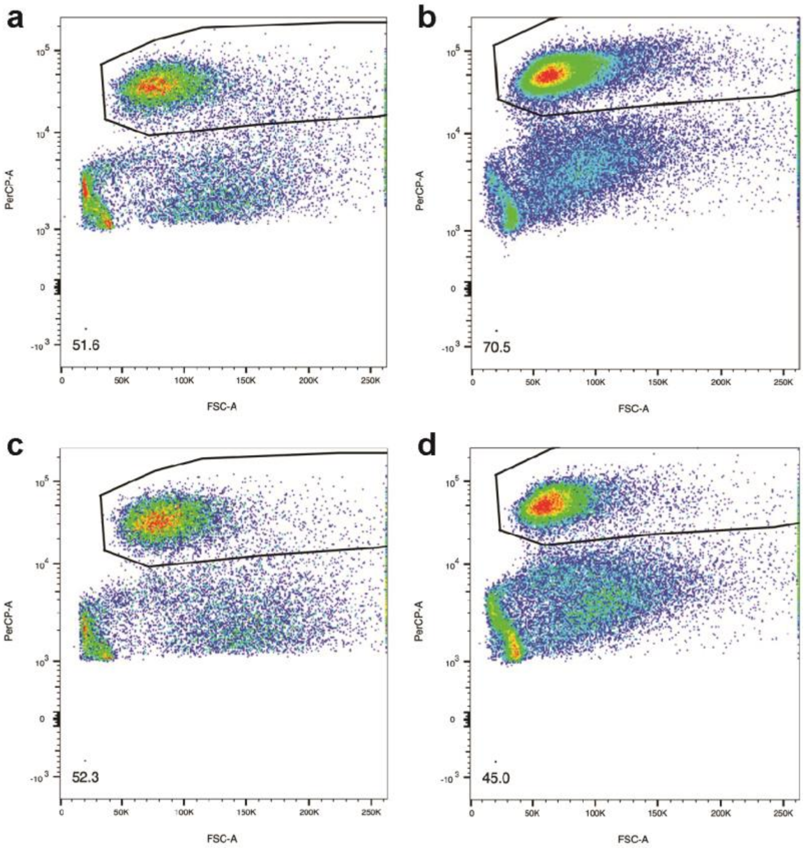

Supplementary Figure 26. Representative images showing gating information in FACS-based symbiont cell counting.

(**a**) Symbiont cell counting for cnidarian animals in control conditions. (**b**) Symbiont cell counting for cnidarian animals in ammonium treatment. (**c**) Symbiont cell counting for cnidarian animals in glucose treatment. (**d**) Symbiont cell counting for cnidarian animals in the combined treatment of glucose and ammonium. Dark black lines in each panel represent gating windows based on forward scatter signals and the chlorophyll fluorescent intensity.

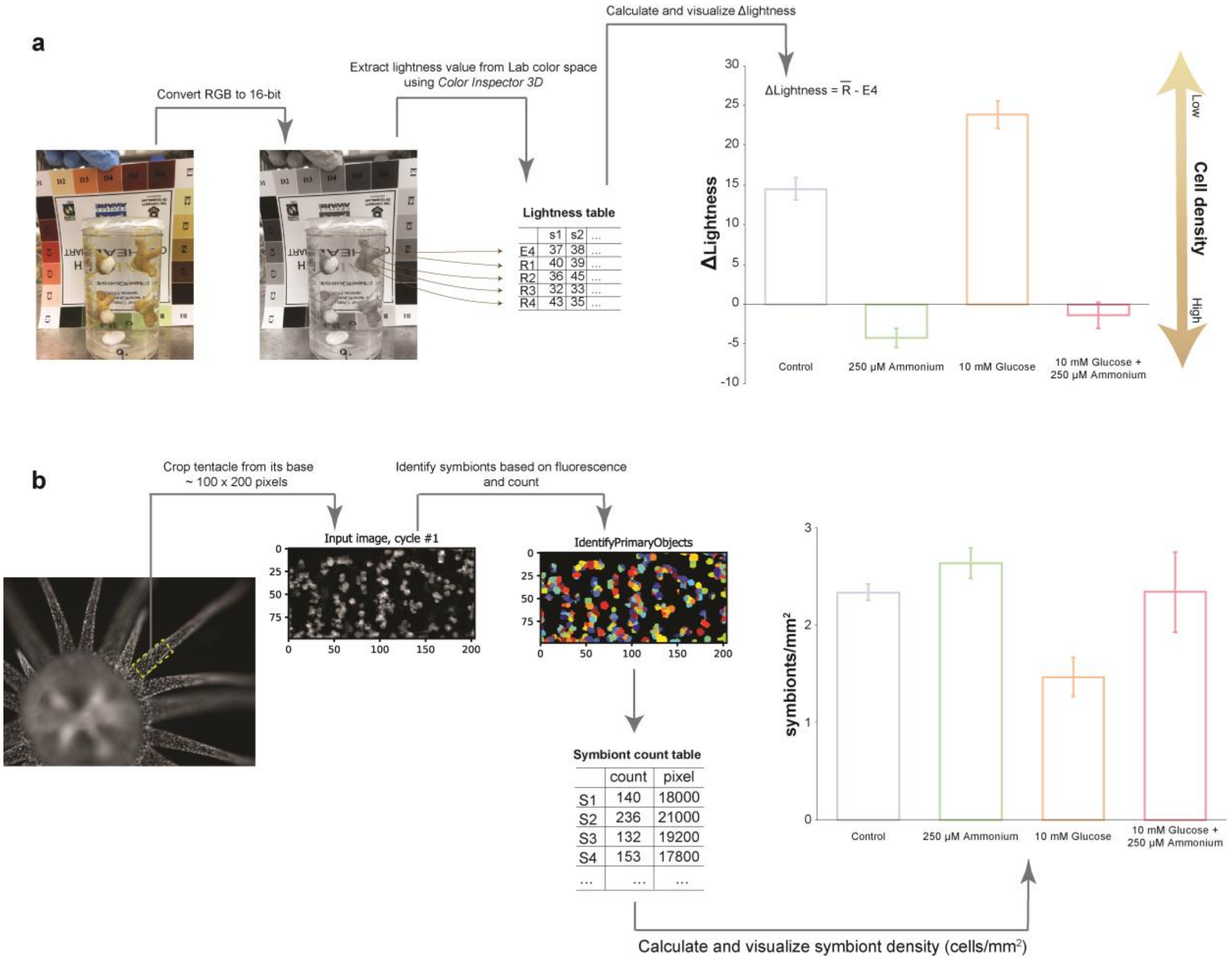

Supplementary Figure 27 Image analysis.

(**a**) Lightness changes in *S. pistillata* fragments, representing symbiont density regulation, analyzed in Fiji using the Color Inspector 3D plugin. (**b**) Symbiont density in the sea anemone *E. diaphana* determined by CellProfiler.

Supplementary Table 1. Deduction of ^{13}C percentage from 3-Phosphohydroxypyruvate and Glycine in cnidarian animals incubated with $^{13}C_6$-glucose

	Average percentage change of ^{13}C	Standard error	Two-sided Welch's *t*-test
Symbiotic *S. pistillata*	55.0%	0.52%	p = 0.0048, *vs*. Symbiotic *C. andromeda*<br>p = 0.0089, *vs*. Symbiotic *E. diaphana*<br>p = 3.4e-7, *vs*. Aposymbiotic *E. diaphana*
Symbiotic *C. andromeda*	25.2%	2.4%	p = 0.0048, *vs*. Symbiotic *S. pistillata*<br>p = 0.98, *vs*. Symbiotic *E. diaphana*<br>p = 0.00055, *vs*. Aposymbiotic *E. diaphana*
Symbiotic *E. diaphana*	25.1%	5.0%	p = 0.0089, *vs*. Symbiotic *S. pistillata*<br>p = 0.98, *vs*. Symbiotic *C. andromeda*<br>p = 0.0013, *vs*. Aposymbiotic *E. diaphana*
Aposymbiotic *E. diaphana*	78.4%	0.93%	p = 3.4e-7, *vs*. Symbiotic *S. pistillata*<br>p = 0.00055, *vs*. Symbiotic *C. andromeda*<br>p = 0.0013, *vs*. Symbiotic *E. diaphana*
