# Supplementary material for: A carbon-nitrogen negative feedback loop underlies the repeated evolution of cnidarian–Symbiodiniaceae symbioses
Source: Nat Commun. 2023 Nov 1;14:6949. doi: 10.1038/s41467-023-42582-y (PMC10620218; doi:10.1038/s41467-023-42582-y)
Supplement: Supplementary file 3 — Description of Additional Supplementary Files [file 41467_2023_42582_MOESM3_ESM.pdf]

Description of Additional Supplementary Files

Supplementary Data 1. Quantitative results of labeled metabolites isolated from symbiotic *S. pistillata*

Supplementary Data 2. Quantitative results of labeled metabolites isolated from symbiotic *E. diaphana*

Supplementary Data 3. Quantitative results of labeled metabolites isolated from symbiotic *C. andromeda*

Supplementary Data 4. Quantitative results of labeled metabolites isolated from aposymbiotic *E. diaphana*

Supplementary Data 5. the relative abundance of isotope-labeled metabolites (13C14N, 12C15N, and 13C15N) normalized to their natural non-labeled forms (12C14N)
