# Supplementary material for: A carbon-nitrogen negative feedback loop underlies the repeated evolution of cnidarian–Symbiodiniaceae symbioses
Source: Nat Commun. 2023 Nov 1;14:6949. doi: 10.1038/s41467-023-42582-y (PMC10620218; doi:10.1038/s41467-023-42582-y)
Supplement: Supplementary file 9 — Reporting Summary [file 41467_2023_42582_MOESM9_ESM.pdf]

nature portfolio

Corresponding author(s): Guoxin Cui, Manuel Aranda

Last updated by author(s): Oct 6, 2023

Reporting Summary

Nature Portfolio wishes to improve the reproducibility of the work that we publish. This form provides structure for consistency and transparency in reporting. For further information on Nature Portfolio policies, see our Editorial Policies and the Editorial Policy Checklist.

Statistics

For all statistical analyses, confirm that the following items are present in the figure legend, table legend, main text, or Methods section.

n/a	Confirmed	
☐	☒	The exact sample size (*n*) for each experimental group/condition, given as a discrete number and unit of measurement
☐	☒	A statement on whether measurements were taken from distinct samples or whether the same sample was measured repeatedly
☐	☒	The statistical test(s) used AND whether they are one- or two-sided<br>*Only common tests should be described solely by name; describe more complex techniques in the Methods section.*
☐	☒	A description of all covariates tested
☐	☒	A description of any assumptions or corrections, such as tests of normality and adjustment for multiple comparisons
☐	☒	A full description of the statistical parameters including central tendency (e.g. means) or other basic estimates (e.g. regression coefficient) AND variation (e.g. standard deviation) or associated estimates of uncertainty (e.g. confidence intervals)
☐	☒	For null hypothesis testing, the test statistic (e.g. *F*, *t*, *r*) with confidence intervals, effect sizes, degrees of freedom and *P* value noted<br>*Give P values as exact values whenever suitable.*
☒	☐	For Bayesian analysis, information on the choice of priors and Markov chain Monte Carlo settings
☒	☐	For hierarchical and complex designs, identification of the appropriate level for tests and full reporting of outcomes
☒	☐	Estimates of effect sizes (e.g. Cohen's *d*, Pearson's *r*), indicating how they were calculated

Our web collection on statistics for biologists contains articles on many of the points above.

Software and code

Policy information about availability of computer code

Data collection	Symbiont count data was collected using a BD LSRFortessaTM Cell Analyzer (BD Biosciences) based on their chlorophyll fluorescence and forward-scatter signals (Supplementary Figure 26).<br>Images of E. diaphana and S. pistillata were collected using a Leica DMI 3000B microscope or a Canon EOS Rebel t5i camera.<br>RNA-seq data was collected by sequencing RNA on an S1 flow cell with the Illumina NovaSeq 6000 platform.<br>Metabolic data was acquired on a Dionex Ultimate 3000 UHPLC system coupled with a Q Exactive Plus mass spectrometer using an ACQUITY UPLC® BEH Amide column.
Data analysis	Coral photos were analyzed using Fiji v2.13.1 (Supplementary Figure 27a). Fluorescent images of E. diaphana were analyzed using CellProfiler v4.2.5 (Supplementary Figure 27b).<br>RNA-seq was analyzed using kallisto v0.44.0 and sleuth v0.29.0. The enrichment analyses were performed with topGO v2.52.0 and clusterProfiler v4.8.1. The enriched GO terms were further summarized and visualized using simplifyEnrichment v1.10.0.<br>Xcalibur v4.2 was used to extract peak areas for the targeted metabolites.<br>base package from R version 4.1.2 was used for the statistical analyses.

For manuscripts utilizing custom algorithms or software that are central to the research but not yet described in published literature, software must be made available to editors and reviewers. We strongly encourage code deposition in a community repository (e.g. GitHub). See the Nature Portfolio guidelines for submitting code & software for further information.

Data

Policy information about availability of data

All manuscripts must include a data availability statement. This statement should provide the following information, where applicable:

- Accession codes, unique identifiers, or web links for publicly available datasets
- A description of any restrictions on data availability
- For clinical datasets or third party data, please ensure that the statement adheres to our policy

Source data are provided with this paper. All quantitative results extracted from UHPLC-HR-MS analysis are provided as Supplementary Data files. The MS raw data generated in this study have been deposited in the NIH Common Fund's National Metabolomics Data Repository under accession code ST002870. RNA-seq data have been deposited in the NCBI Sequence Read Archive under accession codes PRJNA1018325 and PRJNA879277.

Research involving human participants, their data, or biological material

Policy information about studies with human participants or human data. See also policy information about sex, gender (identity/presentation), and sexual orientation and race, ethnicity and racism.

Reporting on sex and gender	Not applicable.
Reporting on race, ethnicity, or other socially relevant groupings	Not applicable.
Population characteristics	Not applicable.
Recruitment	Not applicable.
Ethics oversight	Not applicable.

Note that full information on the approval of the study protocol must also be provided in the manuscript.

Field-specific reporting

Please select the one below that is the best fit for your research. If you are not sure, read the appropriate sections before making your selection.

☒ Life sciences ☐ Behavioural & social sciences ☐ Ecological, evolutionary & environmental sciences

For a reference copy of the document with all sections, see nature.com/documents/nr-reporting-summary-flat.pdf

Life sciences study design

All studies must disclose on these points even when the disclosure is negative.

Sample size	In most of our experiments involving the target animals, three biological replicates typically offer a representative sample. However, we recognize that certain biological parameters can be sensitive to minor changes in the culture environment. Thus, while we generally opt for three biologically independent replicates, we increase this number when feasible to capture as much variance as possible.<br><br>For enzyme activity assays, we consistently used three biological replicates for each condition across all species. For RNA-seq, five replciates were used across all conditions. For cell density measurements, we used nine and five biological replicates for the sea anemone E. diaphana and the jellyfish C. andromeda, respectively. In contrast, the coral species S. pistillata and A. hemprichii had eight replicates each. For targeted metabolomics, the replicates were as follows: four for E. diaphana, three for C. andromeda, and six for S. pistillata. Variations in the number of replicates stemmed from differences in experimental setups for each species or the availability of samples.
Data exclusions	No data was excluded from our analysis.
Replication	All experiments utilized biological replicates. With the exception of the metabolomics experiments and enzyme activity assays, all tests were independently repeated by different researchers, consistently yielding similar results. Due to budgetary constraints, the metabolomics experiments and enzyme activity assays have not been repeated. However, the existing dataset from these tests was analyzed by at least two separate investigators, both of whom arrived at the same conclusions.
Randomization	The allocation of samples in each parallel experiment was randomly applied.
Blinding	No Blinding was conducted. Experiments were performed by at least 3 independent investigators.

Reporting for specific materials, systems and methods

We require information from authors about some types of materials, experimental systems and methods used in many studies. Here, indicate whether each material, system or method listed is relevant to your study. If you are not sure if a list item applies to your research, read the appropriate section before selecting a response.

Materials & experimental systems

n/a	Involved in the study
☒	☐ Antibodies
☒	☐ Eukaryotic cell lines
☒	☐ Palaeontology and archaeology
☐	☒ Animals and other organisms
☒	☐ Clinical data
☒	☐ Dual use research of concern
☒	☐ Plants

Methods

n/a	Involved in the study
☒	☐ ChIP-seq
☐	☒ Flow cytometry
☒	☐ MRI-based neuroimaging

Animals and other research organisms

Policy information about studies involving animals; ARRIVE guidelines recommended for reporting animal research, and Sex and Gender in Research

Laboratory animals	The sea anemone Exaiptasia diaphana strain CC7 were used in the study. All the sea anemones used in experiments share similar size: pedal disc of ~0.5 cm in diameter.<br>Laboratory strain of the jellyfish Cassiopeia andromeda were used in the study. All the jellyfish used in experiments are at similar length of ~1 cm.
Wild animals	Corals Stylophora pistillata and Acropora hemprichii were collected from central the central Red Sea (Al Fahal Reef, 22°14'54" N, 38° 57'46" E). Coral fragments were cut from different colonies, transported back to the lab in water tanks, and acclimatized in indoor tanks for at least three months before experiments.
Reporting on sex	sex of the cnidarian animals in this study is not relevant and has not been assessed.
Field-collected samples	Other than the coral fragments mentioned above, no field-collected samples were used in this study.
Ethics oversight	Experiment involving cnidarian animals do not require ethics oversight.

Note that full information on the approval of the study protocol must also be provided in the manuscript.

Flow Cytometry

Plots

Confirm that:

- ☒ The axis labels state the marker and fluorochrome used (e.g. CD4-FITC).
- ☒ The axis scales are clearly visible. Include numbers along axes only for bottom left plot of group (a 'group' is an analysis of identical markers).
- ☒ All plots are contour plots with outliers or pseudocolor plots.
- ☒ A numerical value for number of cells or percentage (with statistics) is provided.

Methodology

Sample preparation	Symbiont cells were collected using centrifuge following with several rounds of washing and re-pelleting. The final symbiont pellets were then resuspended in appropriate buffer for cell counting using flow cytometry.
Instrument	BD LSRFortessaTM Cell Analyzer
Software	FlowJo was used in gating and basic data extraction.
Cell population abundance	Symbiont cell population takes up to 70% of the sample. It shows clear grouping because of strong native chlorophyll fluorescence.
Gating strategy	Gating was done based on FSC and PerCP-A. PerCP-A signal represent the native chlorophyll fluorescence from symbiont cells.

☒ Tick this box to confirm that a figure exemplifying the gating strategy is provided in the Supplementary Information.
